# Emergent Unilateral Renal Artery Stenting for Treatment of Flash Pulmonary Edema: Fact or Fiction?

**DOI:** 10.1155/2015/659306

**Published:** 2015-02-22

**Authors:** Asaad Akbar Khan, Eugene Patrick McFadden

**Affiliations:** ^1^Cardiology Department, Massachusetts General Hospital, Boston, MA 02114, USA; ^2^Interventional Cardiology Department, Cork University Hospital, Cork, Ireland; ^3^Cork University Hospital, Cork, Ireland

## Abstract

Flash pulmonary edema is characteristically sudden in onset with rapid resolution once appropriate therapy has been instituted (Messerli et al., 2011). Acute increase of left ventricular (LV) end diastolic pressure is the usual cause of sudden decompensated cardiac failure in this patient population. Presence of bilateral renal artery stenosis or unilateral stenosis in combination with a single functional kidney in the susceptible cohort is usually blamed for this condition. We describe a patient who presented with flash pulmonary edema in the setting of normal coronary arteries. Our case is distinct as our patient developed flash pulmonary edema secondary to unilateral renal artery stenosis in the presence of bilateral functioning kidneys. Percutaneous stent implantation in the affected renal artery resulted in rapid resolution of pulmonary edema.

## 1. Introduction

Flash pulmonary edema commonly presents with sudden onset symptoms which typically resolves rapidly (Messerli et al.) [1]. Acute increase of left ventricular (LV) end diastolic pressure is the usual cause of sudden decompensated cardiac failure in this population. It is commonly triggered by acute mitral or aortic regurgitation, myocardial infarction, and sudden decompensation of preexisting heart failure. Rarely, sudden bilateral renal artery occlusion or unilateral stenosis with concurrent single functional kidney has been found causative of this condition.

## 2. Case Report

Our patient is a 76-year-old woman who presented to a peripheral hospital with sudden onset dyspnea, nausea, orthopnea, chest pain, and vague abdominal discomfort. She had a history of vague headaches and a family history of hypertension and ischemic heart disease but did not have a formal diagnosis of hypertension herself. Initial examination and investigations revealed a normal hemoglobin level (12.1 mg/dL), minimally raised neutrophil count, and a normal renal profile. Chest X-ray revealed severe congestive cardiac failure with markedly raised blood pressure (230/130). ECG showed normal sinus rhythm with left ventricular hypertrophic changes which were incorrectly interpreted as left bundle branch block (LBBB). A quick bedside echo revealed moderate left ventricular hypertrophy with a normal left ventricular ejection fraction (LVEF) and no significant valvular lesions (such as mitral or aortic regurgitation or aortic stenosis) which could have accounted for her clinical deterioration. She was commenced on an intravenous nitrate infusion and transferred to the cardiac catheterization laboratory in our tertiary care facility for a presumed acute coronary event leading to decompensated cardiac failure.

On arrival to our facility, the patient was extremely moribund and complained of severe dyspnea and chest discomfort. Her blood pressure was still unchanged at 230/130 mm Hg. Additional antihypertensive pharmacotherapy was considered; however, in view of her unstable clinical status, we decided to shift her to the cardiac catheterization laboratory.

**Figure 1:**
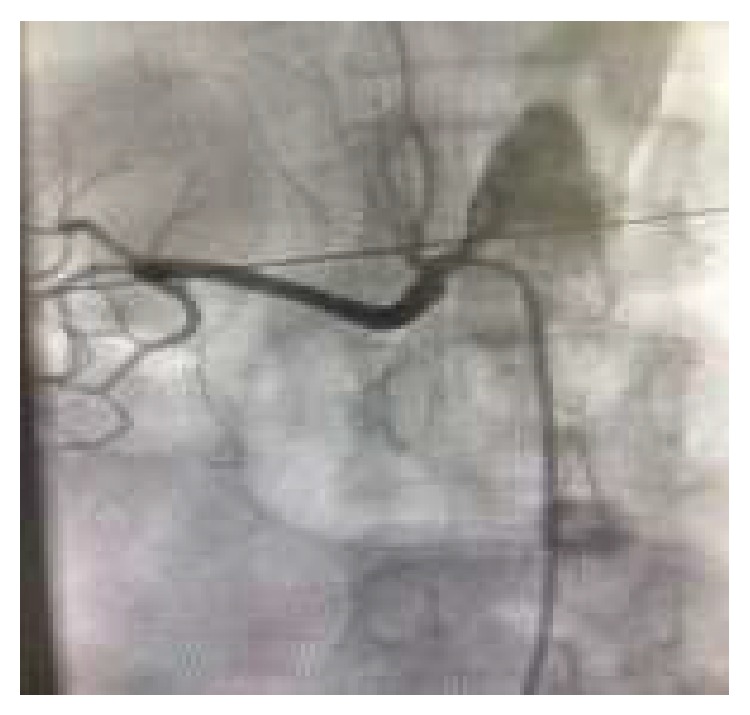
Right renal artery angiogram.

**Figure 2:**
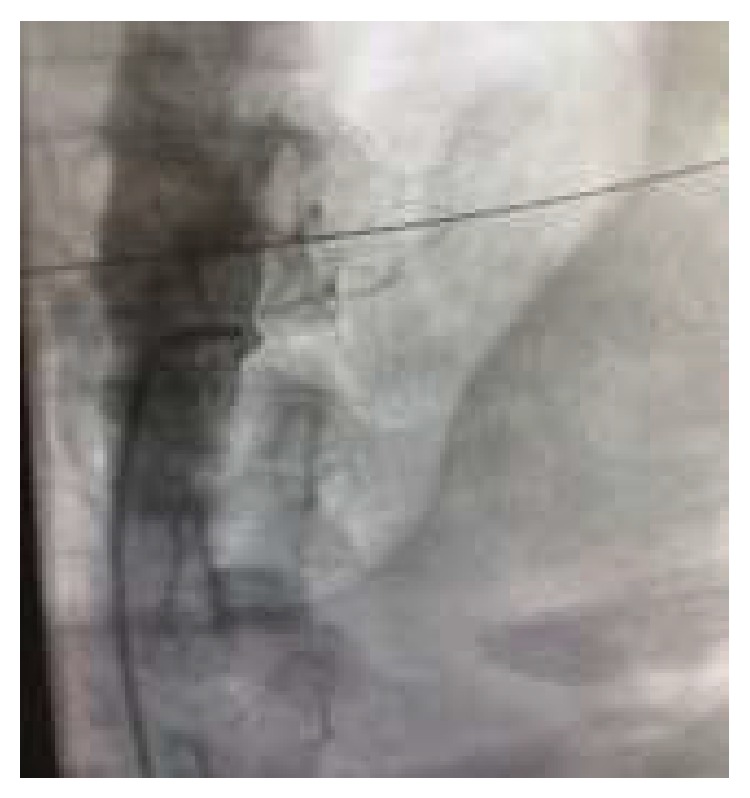
Left renal artery angiogram before intervention.

**Figure 3:**
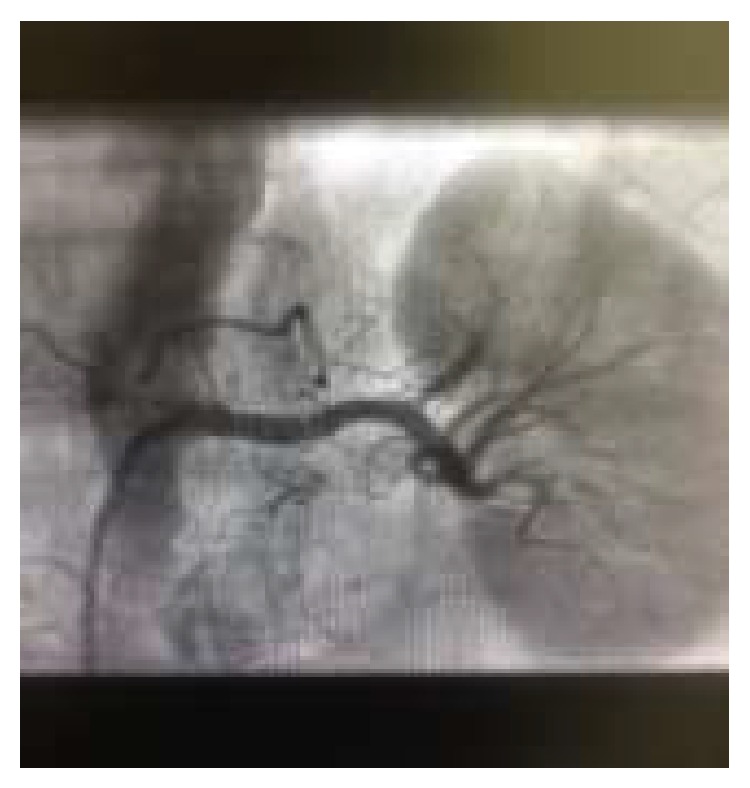
Left renal artery after intervention.

Coronary angiogram revealed nonobstructive coronary artery disease with severely raised end diastolic pressure. In view of the nature of her presentation, renal angiography was performed which showed a normal right renal artery while an ostial occlusion of the left renal artery was noted (Figures 1 and 2). Based on the emergent nature of patient's presentation we decided to proceed with percutaneous intervention (PCI) of the culprit lesion. The left renal artery was reengaged with a JR4 guide catheter and 5000 units of Heparin were administered. The lesion was probed with a Prowater wire whose passage proved difficult; therefore, a 1.25 mm support balloon was used to cross the lesion. Intrarenal position was confirmed by advancing wire into upper and lower pole arteries. This was followed by sequential balloon dilatation with 1.5 mm, 2.5 mm, and 3.0 mm balloons. Intravascular ultrasound (IVUS) was used to assess the lumen of the renal artery. It was found to be a 5.0 mm vessel with severe thrombus load. Thromboaspiration was performed with an export catheter. A Liberte bare metal stent (5.0/12) was inserted and expanded to 16 atm. It was postdilated with a noncompliant balloon to 20 atm and proximal stent edge was flared in the aorta. Multiple injections of isosorbide dinitrate were given (total of 15 mg). IVUS was performed after stenting which revealed appropriate stent size and expansion with good angiographic result (Figure 3). A fractional flow reserve (FFR) wire was used to measure the gradient across the ostial right renal artery which showed a maximum gradient of 10 mmHg. This was consistent with the angiographic data and hence no intervention was performed on the right side.

Blood pressure rapidly normalized and the clinical status of the patient improved after intervention. Patient had a detailed echo the following morning, which confirmed a normal left ventricular ejection fraction, left ventricular hypertrophy with grade 1 diastolic dysfunction, trace to mild MR, left atrial dilation, trace aortic regurgitation, and mild tricuspid regurgitation. An abdominal ultrasound revealed no significant renal abnormalities. Telemetry revealed frequent premature atrial complexes (PACs) and premature ventricular complexes (PVCs) overnight which settled over the following 48 hours. No evidence of atrial fibrillation was noted.

She has been followed up on half yearly basis since and her blood pressure and heart failure control have remained satisfactory. She was prescribed dual antiplatelet therapy for 18 months. A CT aortogram was not performed in her case as atherosclerosis is the commonest cause of renal artery stenosis in people aged >45 years and we felt that further radiation and dye exposure was not warranted.

## 3. Discussion

Atherosclerotic renal artery stenosis is a progressive disease. Sudden progression to complete occlusion of bilateral renal arteries is associated with acute renal failure and sudden onset fluid overload with resultant flash pulmonary edema. Unilateral renal artery stenosis rarely presents with flash pulmonary edema. The exact mechanism of this presentation is not well understood.

Acute renal infarction primarily occurs in patients with other comorbid conditions such as diffuse atherosclerotic disease and atrial fibrillation. These patients typically complain of acute onset of flank or generalized abdominal pain, frequently accompanied by nausea and vomiting. These findings are usually accompanied by an acute elevation in blood pressure that is presumably mediated by increased renin release. Laboratory findings include deterioration in renal indices (creatinine and eGFR), hematuria, and increased LDH [2]. These changes are more pronounced in patients with bilateral disease provided that the contralateral kidney is normal.

Several clinical trials have demonstrated significant improvement in overall renal function in patients with unilateral renal stenosis, following reinstitution of blood flow to the stenotic kidney [3, 4]. One study has also demonstrated reversal of hyperfiltration of the nonstenotic kidney, resulting in decreased proteinuria [5]. These data suggest that in patients with abnormal renal function, treatment of hemodynamically significant unilateral renal artery stenotic lesions can impact favorably on renal function.

La Batide-Alanore et al., showed that bilateral renal artery disease or comparable conditions like unilateral renal artery stenosis with a single functioning kidney, differed from unilateral stenosis with bilateral functioning kidneys in the mechanism by which fluid overload precipitates [6]. Bilateral renal artery stenosis causes pulmonary edema secondary to volume overload. Unilateral renal artery stenosis seems to somehow offset renal angiotensin-II pathway. Following renal artery stenosis, renin is released from the juxtaglomerular (JG) apparatus causing intravascular expansion via sodium and water retention. The ensuing volume overload causes increased left atrial pressure which subsequently results in release of natriuretic peptides leading to effective natriuresis via the normal kidney. However, in bilateral renal artery stenosis, volume overload causes increased left atrial pressure and pulmonary edema as the protective mechanisms are impaired.

Potential pathophysiological mechanisms involved in our patient's presentation are complex and probably do involve the RAS (renin-angiotensin-aldosterone) system. We agree with Noh et al. [7] who have proposed that patients with underlying left ventricular hypertrophy secondary to long standing hypertension have possible diastolic dysfunction and that the left ventricular end diastolic pressures are raised at baseline in this population [8]. The physiology of these noncompliant left ventricles is compromised by sudden small increase in left ventricular end diastolic volume (possibly from a sudden surge in the RAS system) which leads to significantly raised left ventricular end diastolic pressures and subsequent pulmonary edema.

Possible treatment strategies in patients with acute renal artery stenosis include medical, surgical, and percutaneous options [9]. Percutaneous revascularization using angioplasty (PTRA) with or without stenting along with medical therapy has been compared with medical therapy alone in a few major randomized trials and did not confer any significant benefit with respect to the prevention of clinical events when added to comprehensive, multifactorial medical therapy. Some of these trials also included patients with unilateral atherosclerotic renal artery stenosis [10–13]. The importance of renal artery stent placement in the treatment of flash pulmonary edema is not extensively validated and several factors including availability of required equipment and adequate expertise limit its generalization. In a series of patients presenting with either congestive heart failure or an acute coronary syndrome renal artery stent implantation acutely improved symptoms, possibly due to improved neurohormonal and resultant hemodynamic effects [14].

In our patient, the severity of situation and resource availability prompted us towards the percutaneous intervention route which proved successful.

## 4. Conclusion and Future Perspective

Flash pulmonary edema is a rare manifestation of renal artery stenosis. In our patient, it occurred secondary to unilateral renal artery stenosis with bilateral functioning kidneys. Our case demonstrated that unexplained flash pulmonary edema with hypertension should be pursued with a renal angiogram once the coronary artery disease has been outruled. Revascularization in this patient proved that therapeutic intervention with stenting in symptomatic individuals should be performed to reduce morbidity and mortality.
